# Age-dependent relationship between preoperative serum aminotransferase and mortality after cardiovascular surgery

**DOI:** 10.18632/aging.102374

**Published:** 2019-10-18

**Authors:** Jae-Sik Nam, Wook-Jong Kim, Sang-Mee An, Dae-Kee Choi, Ji-Hyun Chin, Eun-Ho Lee, In-Cheol Choi

**Affiliations:** 1 Department of Anesthesiology and Pain Medicine, Laboratory for Perioperative Outcomes Analysis and Research, Asan Medical Center, University of Ulsan College of Medicine, Seoul 05505, Korea

**Keywords:** aging, De Ritis ratio, frailty, liver function tests, transaminase

## Abstract

Although serum aminotransferase levels are frequently measured for preoperative evaluation, their prognostic value to postoperative outcomes remain unclear. This study aimed to investigate the relationship between preoperative serum aminotransferase levels and postoperative 90-day mortality in patients undergoing cardiovascular surgery. We included adult patients (n=6264) who underwent cardiovascular surgery between January 2010 and December 2016 at a tertiary academic hospital. Preoperative serum alanine aminotransferase (ALT), serum aspartate aminotransferase (AST), and De Ritis ratio (defined as AST/ALT) were categorized into three groups: low (≤20th percentile), middle (20th–80th percentile), and high (>80th percentile). Of the 6264 patients enrolled (40.4% women; median age, 62 years), 183 (2.9%) died within 90 days postoperatively. Multivariable-adjusted analyses revealed low ALT (hazard ratio 1.58, 95% confidence interval, 1.14–2.18) and high De Ritis ratio (hazard ratio 1.59, 95% confidence interval, 1.15–2.20) were independent predictors of postoperative mortality, but AST did not have a statistically significant association. The association of low ALT and high De Ritis ratio with 90-day mortality was more pronounced in patients older than 60 years (*P*-values for interaction <0.05). Therefore, preoperative serum aminotransferase levels may be a valuable prognostic marker in patients with cardiovascular surgery, particularly in the elderly.

## INTRODUCTION

Since the discovery of serum aminotransferases in the early 1960s, serum aminotransferase levels, including serum alanine aminotransferase (ALT) and aspartate aminotransferase (AST), have been widely used as indicators of liver function and have also recently gained attention as emerging predictors of cardiovascular disease [1–4].

In general, there have been concerns that elevated serum aminotransferase levels in the preoperative period may be associated with poor postoperative outcomes [5, 6]. This may be partly due to the possibility of hepatocellular damage caused by liver diseases, such as non-alcoholic fatty liver disease, hepatitis, and liver cirrhosis. Furthermore, because of the intimate interaction between the heart and liver [7, 8], elevated preoperative serum aminotransferase levels may be associated with poor postoperative outcomes, especially in patients undergoing cardiovascular surgery.

Conversely, it has been suggested that lower serum aminotransferase activity may be linked to aging-related frailty, reduced liver size, and reduced hepatic blood flow, which could increase vulnerability to various diseases or complications [9–12]. Indeed, several recent studies have reported that low serum aminotransferase levels could be associated with increased mortality, especially in the elderly population [3, 4, 10–12]. Thus, given the growing proportion of elderly patients undergoing cardiovascular surgery, low serum aminotransferase levels may also be associated with adverse postoperative outcomes.

Taken together, both low and high preoperative serum aminotransferase levels may be associated with poor outcomes after cardiovascular surgery. To date, no studies have been conducted to verify this hypothesis. Previous studies have only focused on abnormally elevated serum aminotransferase levels or the De Ritis ratio (defined as the ratio of AST/ALT; proposed by Fernando De Ritis in 1957, the ratio has been used to differentiate liver diseases, such as alcoholic hepatitis) in non-cardiac surgery [6, 13–16]. The objective of the present study was to investigate the associations between preoperative serum AST, ALT, and the De Ritis ratio with survival after cardiovascular surgery and to assess whether the mortality risk associated with preoperative serum aminotransferase levels is age-dependent.

## RESULTS

### Patient characteristics

**Figure 1:**
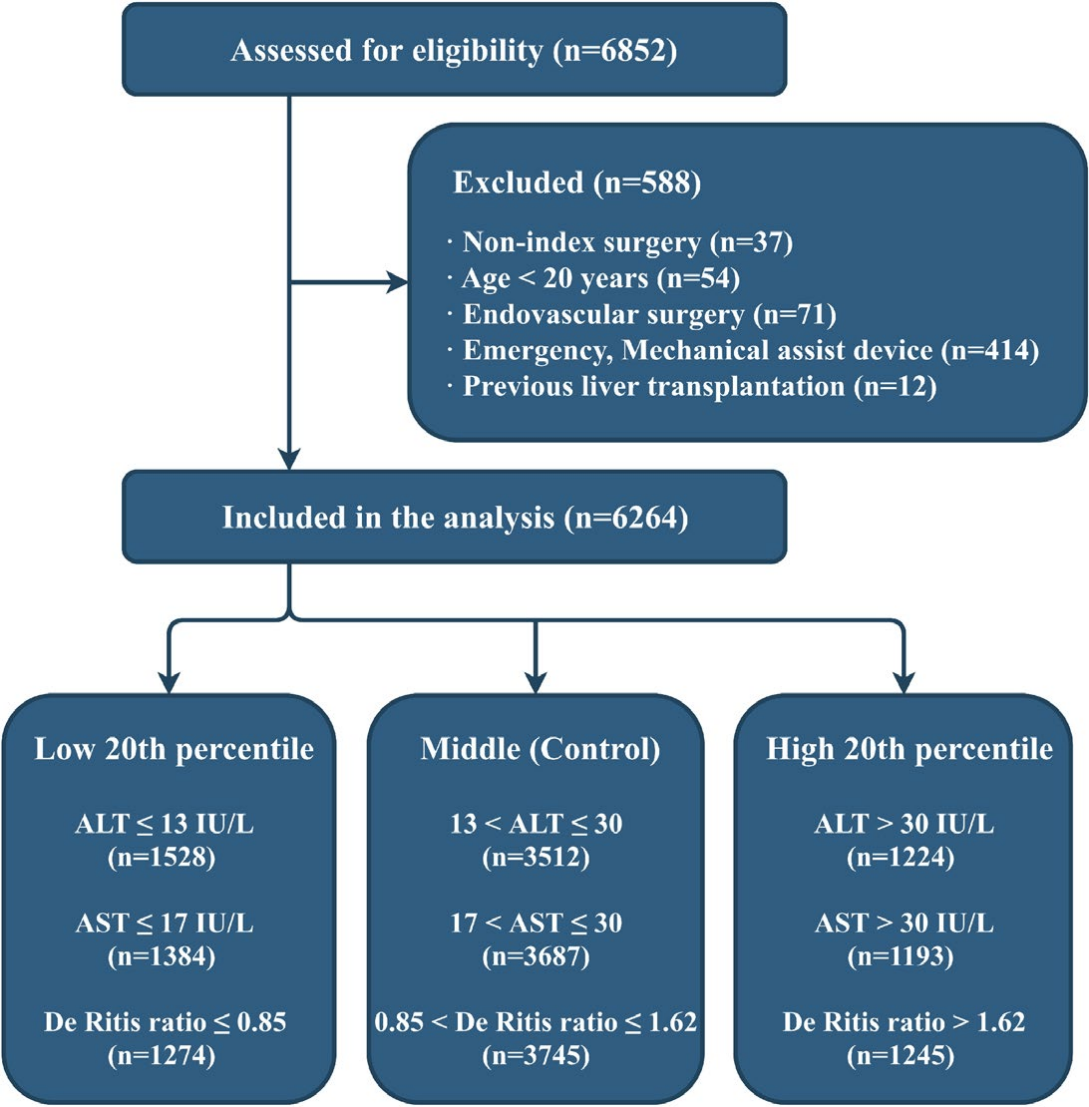
**Flow diagram of the study.** ALT: alanine aminotransferase; AST: aspartate aminotransferase.

Of the 6852 patients assessed for eligibility, 6264 patients were included in the final analysis (Figure 1). The category boundaries of the preoperative serum aminotransferase groups (according to their distribution: low, ≤20th percentile; middle, 20th–80th percentile; high, >80th percentile) are shown in Figure 1.

Table 1 shows the baseline characteristics of the study population. The median age was 62 years (interquartile range, 52–70 years) and 40.4% were women. The patients’ characteristics according to each aminotransferase category are shown in Supplementary Table 1. The age and the proportion of women were significantly higher, and the body mass index was lower for patients in the low ALT category. Patients in the high AST category were more likely to have a history of atrial fibrillation and heart failure and to have received preoperative inotropic or vasopressor agents.

**Table 1:** Patient characteristics.

	**Survivor**	**Non-survivor**	**Total**
*N*	6081	183	6264
Age (years)	61 [52–70]	70 [62–74]	62 [52–70]
Female sex	2444 (40.2)	86 (47.0)	2530 (40.4)
Body mass index (kg/m^2^)	23.9 [21.8–26.1]	23.0 [20.6–25.3]	23.9 [21.7–26.0]
Diabetes mellitus	1476 (24.3)	57 (31.1)	1533 (24.5)
Hypertension	2953 (48.6)	111 (60.7)	3064 (48.9)
Dyslipidemia	4507 (74.1)	117 (63.9)	4624 (73.8)
Congestive heart failure	432 (7.1)	27 (14.8)	459 (7.3)
Coronary revascularization	547 (9.0)	26 (14.2)	573 (9.1)
Liver disease	322 (5.3)	22 (12.0)	344 (5.5)
Alcohol	261 (4.3)	3 (1.6)	264 (4.2)
Acute coronary syndrome	731 (12.0)	22 (12.0)	753 (12.0)
Atrial fibrillation	1296 (21.3)	64 (35.0)	1360 (21.7)
EuroSCORE (logistic)	3.5 [1.9–6.9]	9.8 [4.8–20.2]	3.7 [2.0–7.1]
MELD Xi^*^	9.0 [9.0–11.0]	11.0 [9.0–14.0]	9.0 [9.0–11.0]
Ejection fraction (%)^*^	60.0 [54.0–65.0]	60.0 [48.5–63.5]	60.0 [54.0–65.0]
Pulmonary hypertension^*^	1786 (29.5)	74 (40.4)	1860 (29.8)
Hematocrit (%)	38.9 [35.2–42.0]	34.0 [30.1–38.6]	38.8 [35.0–41.9]
Creatinine (μmol/L)	80 [62–88]	88 [71–124]	80 [62–88]
eGFR (ml/min/1.73 m^2^)	87.1 [70.9–98.3]	68.8 [49.0–86.2]	86.8 [70.2–97.9]
Albumin (g/L)^*^	38 [35–41]	35 [30–38]	38 [35–41]
Bilirubin (μmol/L)^*^	10.3 [6.8–15.4]	12.0 [8.6–18.8]	10.3 [6.8–15.4]
ALT (IU/L)	19 [14–27]	15 [11–21]	19 [14–27]
ALT >40 IU/L	612 (10.1)	11 (6.0)	623 (9.9)
ALT >80 IU/L	96 (1.6)	3 (1.6)	99 (1.6)
AST (IU/L)	22 [18–28]	23 [17–32]	22 [18–28]
AST >40 IU/L	458 (7.5)	27 (14.8)	485 (7.7)
AST >80 IU/L	61 (1.0)	6 (3.3)	67 (1.1)
De Ritis ratio	1.2 [0.9–1.5]	1.5 [1.1–2.0]	1.2 [0.9–1.5]
Sodium (mmol/L)^*^	140 [138–142]	138 [135–141]	140 [138–142]
Uric acid (μmol/L)^*^	327 [262–399]	339 [274–434]	327 [262–399]
C-reactive protein (nmol/L)^*^	0.95 [0.95–3.8]	3.8 [0.95–19]	0.95 [0.95–3.8]
ACEI or ARB	2699 (44.4)	90 (49.2)	2789 (44.5)
β-blocker	2785 (45.8)	82 (44.8)	2867 (45.8)
Calcium channel blocker	2844 (46.8)	93 (50.8)	2937 (46.9)
Diuretics	2628 (43.2)	105 (57.4)	2733 (43.6)
Statin	2900 (47.7)	81 (44.3)	2981 (47.6)
Insulin	930 (15.3)	37 (20.2)	967 (15.4)
Inotropes/vasopressors	198 (3.3)	15 (8.2)	213 (3.4)
Type of surgery			
Coronary artery bypass	1704 (28.0)	27 (14.8)	1731 (27.6)
Valve	2717 (44.7)	66 (36.1)	2783 (44.4)
Aorta	370 (6.1)	29 (15.8)	399 (6.4)
Other	267 (4.4)	4 (2.2)	271 (4.3)
Combined	1023 (16.8)	57 (31.1)	1080 (17.2)
Urgent surgery	177 (2.9)	15 (8.2)	192 (3.1)
Off-pump surgery	1456 (23.9)	17 (9.3)	1473 (23.5)
Operation time (min)	270 [220–339]	389 [302–523]	273 [221–343]
CPB time (min)	111 [46–158]	183 [109–250]	112 [50–161]

Data are expressed as the number of patients (%) and mean (± standard deviation) or median [interquartile range].

^*^Variables with missing values: Ejection fraction (5/6264; 0.08%), pulmonary hypertension (28/6264; 0.45%), albumin (1/6264; 0.02%), bilirubin (1/6264; 0.02%), sodium (1/6264; 0.02%), uric acid (2/6264; 0.03%), C-reactive protein (295/6264; 4.71%), and MELD Xi (1/6264; 0.02%).

SI conversion factors: To convert ALT and AST to μkat/L, multiply values by 0.0167.

EuroSCORE: The European System for Cardiac Operative Risk Evaluation; MELD Xi: Model for End-stage Liver Disease eXcluding INR; eGFR: estimated glomerular filtration rate; ALT: alanine aminotransferase; AST: aspartate aminotransferase; ACEI: angiotensin-converting enzyme inhibitor; ARB: angiotensin receptor blocker; CPB: cardiopulmonary bypass.

### Age-dependent relationship between serum aminotransferase and postoperative mortality

The survival status of all patients was completely identified 90 days postoperatively. Postoperative outcomes are shown in Table 2. Of the 6264 patients, 183 (2.92%) died within 90 days postoperatively (median time to death, 17 days [interquartile range, 5–38 days]). Ninety-day mortality rates were higher for patients in the low ALT category (5.0% *vs.* 2.3% *vs.* 2.1%; *P<*0.001), high AST category (3.3% *vs.* 2.3% *vs.* 4.4%; *P=*0.001), and high De Ritis ratio category (1.3% *vs.* 2.3% *vs.* 6.3%; *P<*0.001).

**Table 2:** Postoperative outcomes in the study groups.

	**ALT**			**AST**			**Total**
	**Low**	**Middle**	**High**	**Low**	**Middle**	**High**	
*N*	1528	3512	1224	1384	3687	1193	6264
90-day death	77 (5.0)	80 (2.3)	26 (2.1)	46 (3.3)	85 (2.3)	52 (4.4)	183 (2.9)
In-hospital death	73 (4.8)	80 (2.3)	26 (2.1)	42 (3.0)	85 (2.3)	52 (4.4)	179 (2.9)
Prolonged hospital length of stay	399 (26.1)	714 (20.3)	250 (20.4)	279 (20.2)	754 (20.5)	330 (27.7)	1363 (21.8)
Major adverse cardiovascular events	80 (5.2)	148 (4.2)	52 (4.2)	52 (3.8)	143 (3.9)	85 (7.1)	280 (4.5)
Stroke	49 (3.2)	72 (2.1)	25 (2.0)	41 (3.0)	73 (2.0)	32 (2.7)	146 (2.3)
Pulmonary complications	202 (13.2)	316 (9.0)	97 (7.9)	139 (10.0)	339 (9.2)	137 (11.5)	615 (9.8)
Acute kidney injury	181 (11.8)	288 (8.2)	80 (6.5)	128 (9.2)	297 (8.1)	124 (10.4)	549 (8.8)
Gastrointestinal complications	20 (1.3)	32 (0.9)	12 (1.0)	13 (0.9)	40 (1.1)	11 (0.9)	64 (1.0)

Data are expressed as the number of patients (%).

ALT: alanine aminotransferase; AST: aspartate aminotransferase.

**Figure 2:**
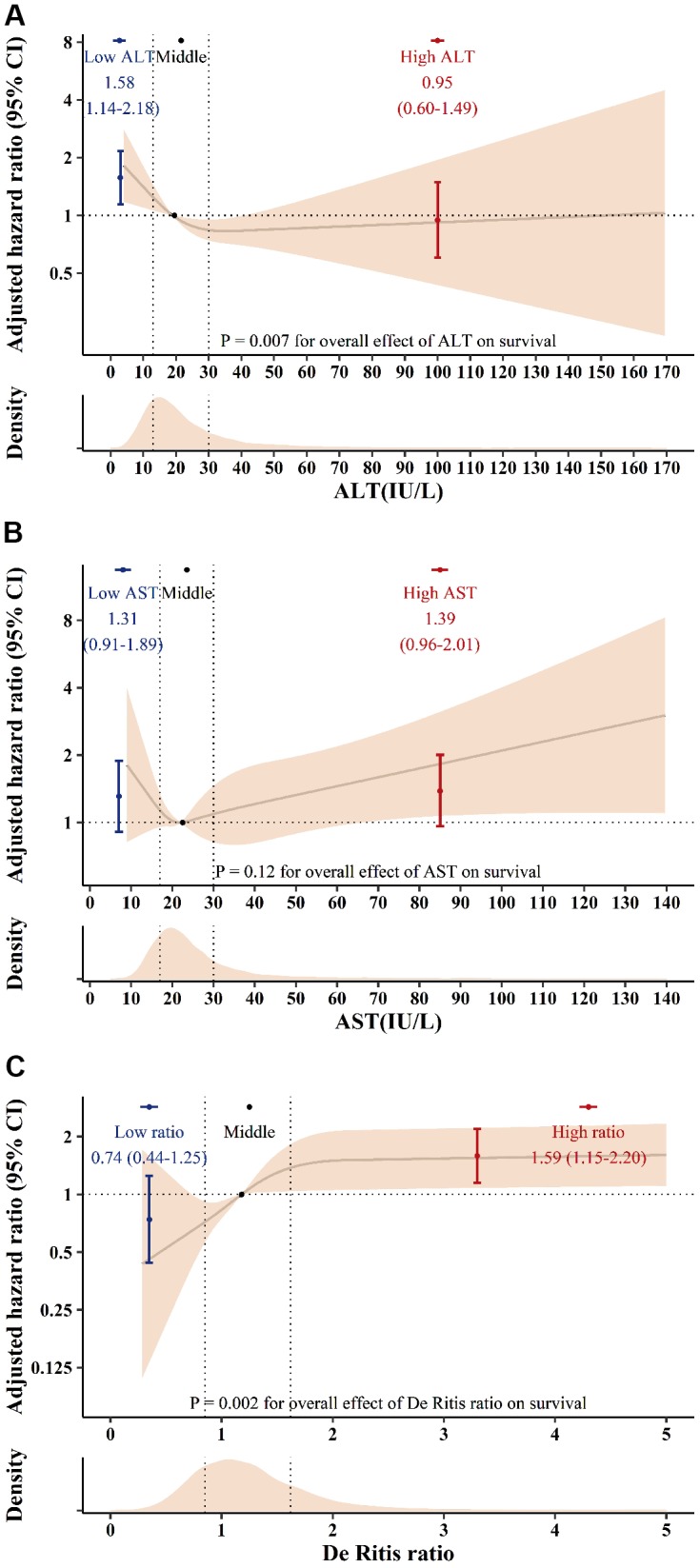
The adjusted hazard ratio for ALT (**A**), AST (**B**) and the De Ritis ratio (**C**) for postoperative 90-day mortality. Solid lines indicate the hazard ratio according to ALT, AST, and De Ritis ratio splines with medians (19 IU/L for ALT, 22 IU/L for AST and 1.18 for the De Ritis ratio) as a reference. The shaded area represents the 95% confidence interval. Dots indicate the hazard ratio according to ALT, AST, and De Ritis ratio groups. Error bars represent the 95% confidence intervals. SI conversion factors: To convert ALT and AST to μkat/L, multiply values by 0.0167. ALT: alanine aminotransferase; AST: aspartate aminotransferase; CI: confidence interval.

Univariate analysis revealed low preoperative ALT and high De Ritis ratio categories were associated with anincreased risk of 90-day mortality. In the AST, both low and high categories were associated with an increased risk of 90-day mortality (Table 3). After adjustments in the multivariable analyses (included variables: sex, age, body mass index, estimated glomerular filtration rate, type of surgery, urgent surgery, ejection fraction, pulmonary hypertension, acute coronary syndrome, liver disease, statin, alcohol consumption, albumin, and bilirubin), the low ALT and high De Ritis ratio categories were determined to be independently associated with increased 90-day mortality risk (Table 3 and Figure 2). Although preoperative AST appeared to have a U-shaped hazard function for postoperative mortality, there were no statistically significant associations between AST and 90-day mortality (Figure 2).

**Table 3:** Impact of preoperative serum aminotransferase levels on 90-day mortality.

	**Death**	**Patients**	**Cumulative mortality**	**Unadjusted**	**Multivariable adjusted**
	No.	No.	Rate (95% CI)	Hazard ratio (95% CI)	Hazard ratio (95% CI)
ALT					
Low (≤13 IU/L)	77	1528	5.0 (3.9–6.1)	2.25 (1.65–3.08)^‡^	1.58 (1.14–2.18)†
Middle (13–30 IU/L)	80	3512	2.3 (1.8–2.8)	1 (reference)	1 (reference)
High (>30 IU/L)	26	1224	2.1 (1.3–2.9)	0.93 (0.60–1.45)	0.95 (0.60–1.49)
AST					
Low (≤17 IU/L)	46	1384	3.3 (2.4–4.3)	1.45 (1.02–2.08)^*^	1.31 (0.91–1.89)
Middle (17–30 IU/L)	85	3687	2.3 (1.8–2.8)	1 (reference)	1 (reference)
High (>30 IU/L)	52	1193	4.4 (3.2–5.5)	1.91 (1.35–2.70)^‡^	1.39 (0.96–2.01)
De Ritis ratio					
Low (≤0.85)	17	1274	1.3 (0.7–2.0)	0.57 (0.34–0.96)^*^	0.74 (0.44–1.25)
Middle (0.85–1.62)	87	3745	2.3 (1.8–2.8)	1 (reference)	1 (reference)
High (>1.62)	79	1245	6.3 (5.0–7.7)	2.79 (2.05–3.78)^‡^	1.59 (1.15–2.20)^†^

^*^P<0.05, ^†^*P<*0.01, ^‡^*P<*0.001.

SI conversion factors: To convert ALT and AST to μkat/L, multiply values by 0.0167.

ALT: alanine aminotransferase; AST: aspartate aminotransferase; CI: confidence interval.

**Figure 3:**
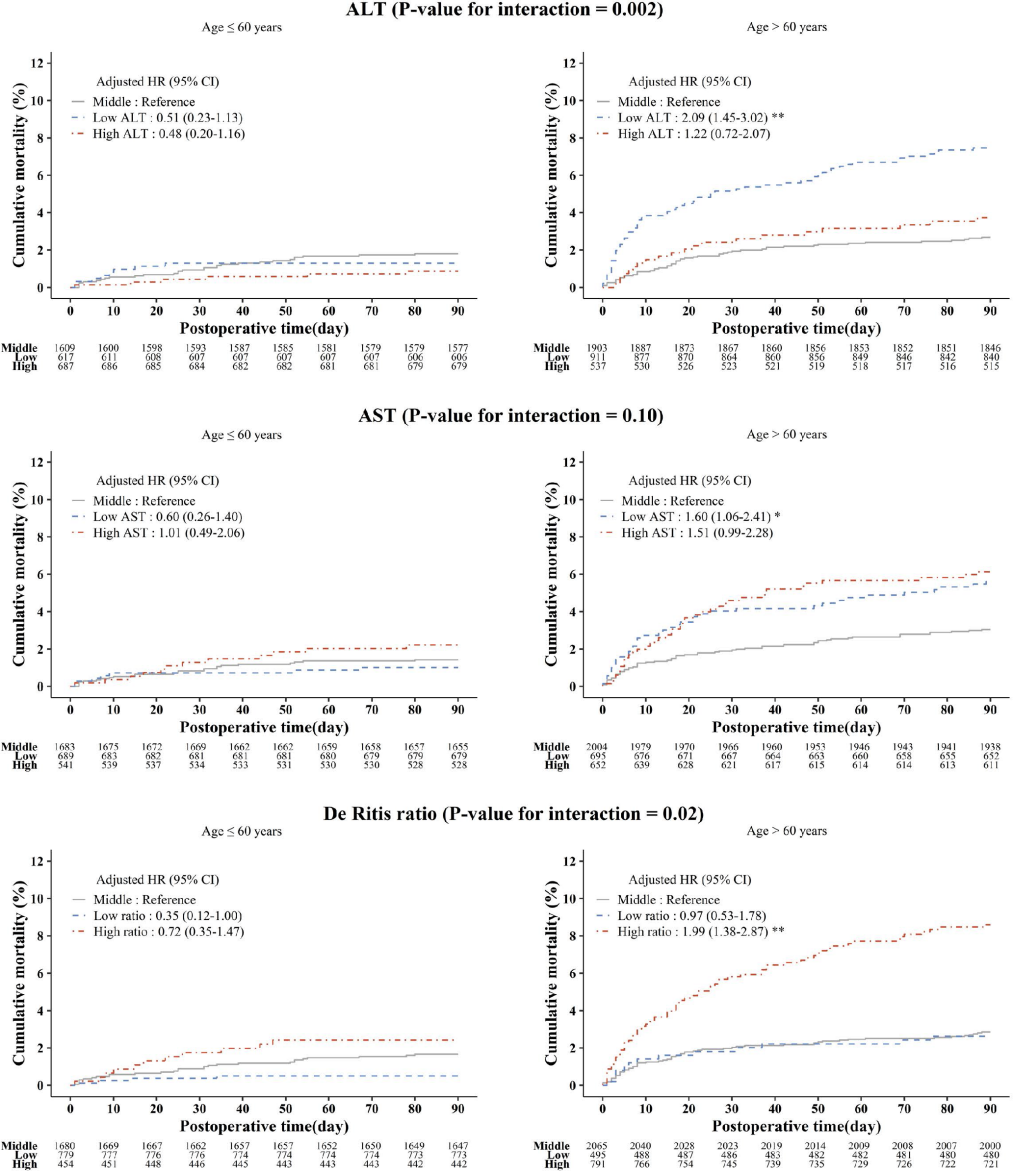
**Kaplan-Meier estimated postoperative 90-day cumulative mortality and adjusted hazard ratios according to the ALT, AST, and De Ritis ratio groups stratified by age.** Asterisks indicate the statistical significance. ^*^ *P<*0.05, ^**^ *P<*0.001. ALT: alanine aminotransferase; AST: aspartate aminotransferase; HR: hazard ratio; CI: confidence interval.

The relationship between low ALT and high De Ritis ratio categories with 90-day mortality was more pronounced in patients older than 60 years (Figure 3). However, interaction between aminotransferase levels and liver disease were not statistically significant (*P=*0.96 for ALT, *P=*0.35 for AST, and *P=*0.44 for De Ritis ratio).

### Incremental value of serum aminotransferase as a prognostic marker

The addition of preoperative serum ALT and De Ritis ratio into the multivariable model significantly improved the model’s predictive ability (net reclassification improvement: 17.81% [95% confidence interval (CI) 5.95%–25.06%, *P*<0.001] for ALT, 15.19% [95% CI 3.02%–26.28%, *P*=0.01] for De Ritis ratio; integrated discrimination improvement: 0.42% [95% CI 0.07%–1.38%, *P*<0.01] for ALT, 0.62% [95% CI 0.21%–1.66%, *P*<0.001] for De Ritis ratio). In contrast, preoperative AST provided less incremental prognostic information beyond clinical risk factors (net reclassification improvement, 12.84% [95% CI -3.19%–20.04%, *P*=0.09]; integrated discrimination improvement, 0.24% [95% CI 0.00%–1.18%, *P*=0.04]).

### Association with secondary outcomes and sensitivity analysis

**Figure 4:**
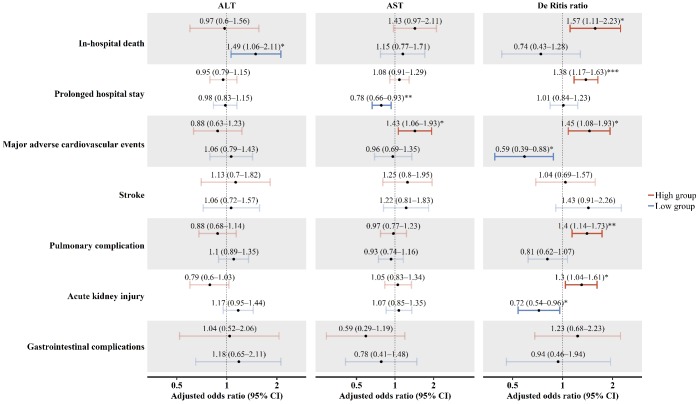
**Secondary outcomes.** Dots indicate the adjusted odds ratio of secondary outcomes according to the ALT, AST, and De Ritis ratio groups. Error bars represent the 95% confidence intervals. Asterisks indicate the statistical significance. ^*^ *P<*0.05, ^**^ *P<*0.01, ^***^ *P<*0.001. ALT: alanine aminotransferase; AST: aspartate aminotransferase; CI: confidence interval.

Figure 4 shows the adjusted associations between aminotransferase levels and exploratory secondary outcomes. The preoperative low ALT group was associated with a higher risk for in-hospital mortality. The preoperative high AST group was associated with postoperative major adverse cardiovascular events. The high De Ritis group was associated with a higher risk of in-hospital mortality, prolonged hospital length of stay, major adverse cardiovascular events, pulmonary complications, and acute kidney injury. Our findings were preserved across the sensitivity analyses (Table 4).

**Table 4:** Sensitivity analysis.

	**Sensitivity analysis I^‡^**		**Sensitivity analysis II^§^**		**Sensitivity analysis III^||^**	
	**Boundary**	**HR (95% CI)**	**Boundary**	**HR (95% CI)**	**Boundary**	**HR (95% CI)**
ALT (IU/L)						
Low	≤11	1.43 (1.01–2.02)^*^	≤9	1.20 (0.79–1.84)	≤13	1.69 (1.23–2.34)^†^
Middle		1 (reference)		1 (reference)		1 (reference)
High	>35	0.88 (0.54–1.44)	>23	0.68 (0.47–0.99)^*^	>30	0.95 (0.61–1.48)
AST (IU/L)						
Low	≤16	1.32 (0.89–1.95)	≤17	1.39 (0.90–2.14)	≤17	1.35 (0.93–1.96)
Middle		1 (reference)		1 (reference)		1 (reference)
High	>34	1.42 (0.96–2.09)	>22	1.27 (0.86–1.86)	>30	1.38 (0.97–1.97)
De Ritis ratio						
Low	≤0.78	0.57 (0.30–1.10)	≤1.19	0.63 (0.44–0.91)^*^	≤0.85	0.58 (0.34–0.99)^*^
Middle		1 (reference)		1 (reference)		1 (reference)
High	>1.75	1.67 (1.21–2.32)^†^	>2.06	1.54 (1.05–2.26)^*^	>1.62	1.59 (1.15–2.21)^†^

^*^P<0.05, ^†^*P<*0.01.

^‡^Serum aminotransferase levels were categorized into ≤15th percentile, 15th–85th percentile, and >85th percentile.

^§^Serum aminotransferase levels were categorized by “K-Adaptive Partitioning for survival data” method.

^‡ §^Adjusted by sex, age, body mass index, estimated glomerular filtration rate, type of surgery, urgent surgery, ejection fraction, pulmonary hypertension, acute coronary syndrome, albumin, bilirubin, liver disease, statin, and alcohol.

^||^Adjusted with EuroSCORE, MELD Xi, diabetes mellitus, hypertension, heart failure, coronary revascularization, atrial fibrillation, hematocrit, sodium, uric acid, diuretics, C-reactive protein, inotropes/vasopressors, dyslipidemia, alcohol, albumin, and liver disease.

SI conversion factors: To convert ALT and AST to μkat/L, multiply values by 0.0167.

ALT: alanine aminotransferase; AST: aspartate aminotransferase; HR: hazard ratio; CI: confidence interval.

## DISCUSSION

### Key findings

This observational study of 6264 patients who underwent cardiovascular surgery showed that after adjusting for potential confounders, a low preoperative serum ALT level and high De Ritis ratio were associated with increased postoperative 90-day mortality, but preoperative AST levels were not. Furthermore, the relationships of preoperative ALT and De Ritis ratio to postoperative mortality were modified by age, with more pronounced associations in older patients (>60 years). ALT and De Ritis ratio also provided incremental values for risk prediction beyond clinical risk factors.

### Interpretation

The negative correlation between the preoperative serum ALT level and postoperative mortality may be contrary to most clinicians’ expectations and seems paradoxical. ALT is a liver-specific enzyme, so serum ALT elevation is a predominant marker of liver cell damage, which has been postulated to have a negative impact on postoperative outcome. Surprisingly, our results suggest that postoperative survival was not worse in patients with high preoperative ALT, but was worse in those with low ALT. This is consistent with the results of previous studies of the general population. The inverse relationship between serum ALT and mortality was first reported in 2006 by Elinav et al. [10], and subsequent studies validated this concept in different cohorts [4, 11, 12, 17–19]. Meta-analyses also confirmed that low serum ALT levels were associated with increased overall and cardiovascular mortality in older populations [2, 3]. Similar results were reported in patients with heart failure [20], diabetes [21], and previous myocardial infarction or stroke [4]. The exact mechanism involved in the relationship between low serum ALT levels and increased mortality has not yet been elucidated. However, previous researchers have postulated that low ALT levels might be a marker of hepatic aging and frailty, since low ALT is more strongly associated with risk of death in the elderly [2, 3]. Serum ALT was substantially lower in older patients, and low ALT may reflect hepatic aging, which accompanies a decrease in liver volume and functional liver cells [10, 11, 22]. Furthermore, decreased ALT levels were associated with frailty as defined by Fried’s frailty criteria and a frailty questionnaire score (FRAIL scale: Fatigue, Resistance, Ambulation, Illnesses, and Loss of Weight) [11, 12, 23]. A recent study also showed that low ALT levels were linked with sarcopenia, a common condition in frail patients [12]. Our results showed the association of ALT with postoperative mortality was more significant in older patients. Hence, it may be plausible that low ALT could be a marker of frailty and liver aging. Another possible explanation is that low ALT may represent poor nutritional status [10, 12, 20]. Vitamin B_6_ deficiency, which is commonly accompanied by malnutrition, decreases serum ALT levels by reducing hepatic ALT synthesis [24]. In addition, vitamin B_6_ deficiency results in further lowering the detected level of serum ALT, as the serum ALT assay requires sufficient vitamin B_6_ in the patient’s serum as a cofactor [24]. Another noteworthy result from our study was that low ALT predicted postoperative mortality, despite the lack of significant associations with other specific major adverse events. Thus, low ALT levels may reflect a diminished physiological reserve to recover from postoperative insults, rather than predicting certain complications.

Similarly, AST appears to have an inverse association with postoperative mortality at low levels. Although the available literature related to ALT is more extensive, the relationship between low AST and increased mortality has been reported in previous studies [4, 18]. Decreased survival may be due to hepatic aging and patient vulnerability, as observed in patients with low ALT. Conversely, high AST levels showed a positive association with postoperative mortality. Considering the low liver specificity of AST and the low mortality rate in the high ALT group from our results, hepatic disease may not be the cause of the increased mortality observed in the high AST level group. Indeed, AST is one of the cardiac markers [25], and elevated AST levels may indicate a hypoperfusion state due to cardiac dysfunction [8, 20]. Thus, the increased mortality associated with high AST levels may result from underlying cardiac dysfunction and myocardial damage. The increased number of major adverse cardiovascular events in the high AST group may support this explanation. However, the U-shaped relationship between preoperative AST and postoperative mortality was not significant after adjustment with possible confounders. Other known risk factors in cardiovascular surgery may act as confounders due to the organ non-specificity of AST. Therefore, given AST’s lack of organ specificity and the availability of more specific markers, such as troponin I, creatine kinase-muscle/brain, and ALT, the usefulness of AST alone as a predictor of postoperative mortality in cardiovascular surgery may be inadequate.

Finally, the De Ritis ratio showed a positive independent association with postoperative mortality. It seems plausible because ALT has a negative correlation with postoperative mortality and the increasing trend of mortality was observed in the high AST group. This result is in line with previous studies reporting the De Ritis ratio as a predictor of mortality after urologic cancer surgery [13, 14]. However, previous studies have only focused on the numerator of the ratio and explained that the increased De Ritis ratio reflects increased AST activity resulting from anaerobic metabolism of cancer cells. Considering the previously described importance of low ALT, the De Ritis ratio should also be interpreted with regard to the denominator. Taken together, the De Ritis ratio may be a composite marker that reflects cardiac status for a given patient’s vulnerability and may be a useful predictor of postoperative mortality and morbidity in cardiovascular surgery.

### Clinical implications

First, our results suggest new insights into the preoperative serum aminotransferase levels in cardiovascular surgery. Most perioperative clinical interest has focused on elevated serum aminotransferase levels. Nearly all clinicians often consider normal serum aminotransferase levels to reflect a healthy liver, but this may not be true. Our results suggest that clinicians should pay closer attention to low ALT levels and high De Ritis ratios in patients requiring cardiovascular surgery, particularly in the elderly. As the number of elderly individuals requiring cardiovascular surgery increases, determining the most appropriate intervention for older patients has become a critical clinical issue. To gain further insight into this issue, interest in biological age, rather than chronologic age, and frailty of the elderly has been increasing. Several scoring systems and assessment tools have been proposed, but the expense of time and resources makes it difficult to utilize these methods with all surgical procedure candidates [26]. Thus, there has been a demand to develop a simple method to assess frailty, such as using ICD-10 codes [27]. In this respect, the use of serum aminotransferase levels has the advantage of being easy to evaluate at a low cost; it may become a relevant risk factor that reflects hepatic aging and overall frailty in cardiovascular surgery. However, this concept should be validated in further studies under different settings.

Second, mild serum aminotransferase elevation appears to have a limited association with poor postoperative prognosis in cardiovascular surgery. Thus, delaying surgery to perform an additional evaluation of the liver may be not necessary for patients asymptomatic of liver disease. Indeed, it has been recommended that patients can proceed to surgery without additional tests if AST and ALT levels are less than twice the upper normal limit [28]. However, this recommendation was not based on high-quality evidence [5]. Others have recommended postponing elective surgery until the determination of the etiology or resolution of the elevated aminotransferase [29]. The results of our study, the comparable prognosis of high ALT with that of normal range, may assist clinicians in determining patients’ prognosis and reduce wasted resources, overspending, and patient anxiety. However, careful review of medical history and physical examinations should always be a requirement in patients with elevated aminotransferase.

### Limitations

First, we cannot provide a precise mechanism for the relationship between preoperative serum aminotransferase and postoperative mortality due to the retrospective study design. Although we used nearly 30 variables available in our database for adjustment, some possible confounders, including liver volume, vitamin B_6_ level, nutritional status, and sarcopenia, could not be collected. Therefore, further studies are required to explore underlying mechanisms, especially modifiable ones, such as vitamin B_6_ deficiency and poor nutritional status.

Second, it is unclear whether our results may be generalized for patients whose preoperative serum aminotransferase levels are more than twice the upper normal limit. Indeed, we anticipated performing additional sensitivity analyses for aminotransferase levels more than twice the upper normal limit. However, even after the inclusion of 6264 patients, the number of patients with such high preoperative serum aminotransferase levels was less than 100 (1.6%). Thus, it was difficult to precisely analyze risk in these groups, and the confidence interval in the continuous analysis was also very wide in this range. Furthermore, not all patients with aminotransferase levels more than twice the upper normal limit proceeded to surgery, and some patients underwent the procedure only after normalization of aminotransferase levels. This may represent a selection bias. Therefore, our results should be interpreted with caution for preoperative serum aminotransferase levels above twice the upper normal limit.

## CONCLUSIONS

In patients undergoing cardiovascular surgery, low preoperative serum ALT levels and high De Ritis ratios were significantly associated with postoperative 90-day all-cause mortality, particularly in the elderly. Therefore, preoperative serum aminotransferase levels may be a valuable prognostic marker in cardiovascular surgery. These findings should be validated in further studies.

## MATERIALS AND METHODS

### Study participants

This observational cohort study included patients who underwent cardiovascular surgery between January 2010 and December 2016 at a tertiary academic hospital in South Korea. The Institutional Review Board (AMC IRB 2019-0176) approved this study protocol and waived the requirement for informed consent owing to the anonymous and retrospective nature of the study. This study was carried out in accordance with the Declaration of Helsinki and the Strengthening the Reporting of Observational Studies in Epidemiology guidelines [30].

All clinical information on the study population was collected from the Electronic Medical Record System (Asan Medical Center Information System Electronic Medical Record) and the Asan Medical Center Cardiovascular Surgery and Anesthesia Database [31]. All patients in the database were assessed for eligibility. For patients who underwent more than one operation during a single hospital stay, the first scheduled operation was considered as the index procedure, and the non-index procedures were excluded. Patients under 20 years of age, those who underwent endovascular surgery or emergency surgery, those with a preoperative intra-aortic balloon pump or ventricular assist device support, and those who previously underwent liver transplantation were excluded.

### Definitions of study exposures, outcomes, and variables

As we hypothesized that both low and high preoperative serum aminotransferase levels might associate with poor postoperative outcomes, serum aminotransferase levels were categorized into three groups according to their distribution: low (≤20th percentile; exposure), middle (20th–80th percentile; control), and high (>80th percentile; exposure). We also performed continuous analysis to evaluate the dose-response relationship between serum aminotransferase levels and the primary outcome. The primary outcome was reported as postoperative 90-day all-cause mortality according to recent recommendations from the European Society of Anaesthesiology and the European Society of Intensive Care Medicine [32]. The survival status of patients was obtained from our medical records or the National Health Insurance status. Exploratory secondary outcomes were in-hospital death, prolonged hospital length of stay (>14 days), major adverse cardiovascular events, stroke, pulmonary complications, acute kidney injury, and gastrointestinal complications within 30 days postoperatively. Detailed definitions of secondary outcomes and variables are described in Supplementary Table 2.

### Perioperative management and laboratory tests

Our perioperative management was carried out in accordance with the standards of our institution as previously described [31, 33]. As part of the preoperative evaluation, all patients underwent routine chemistry tests using the Roche cobas 8000 c702 analyzer (Roche Diagnostics, Mannheim, Germany) at the central laboratory. These tests included the measurement of serum ALT and AST levels as well as other crucial prognostic biomarkers, including creatinine and albumin. Hepatitis B virus surface antigen and hepatitis C virus antibodies were also routinely screened. In our laboratory, the serum aminotransferase levels were measured using the modified International Federation of Clinical Chemistry method without pyridoxal-5’-phosphate supplement. The AST and ALT threshold of 40 IU/L (0.67 μkat/L in SI units) was used independently of patient sex. The patients with borderline elevation of preoperative serum aminotransferase levels (defined as less than twice the upper normal limit) underwent scheduled surgery without any further evaluation unless there were symptoms and signs of liver disease. For patients with serum aminotransferase levels more than twice the upper normal limit, a multidisciplinary and individualized management plan was devised.

### Statistical analysis

The sample size was initially driven by all eligible patients and followed by statistical power analysis. Assuming an overall postoperative 90-day mortality of 2.9% [34] and a clinically significant difference of 2% to detect in exposure groups, a two-sided log-rank test with a sample size of 6264 subjects could achieve 93.5% power at a 0.05 significance level. For missing values, single value imputation was performed using the Markov chain Monte Carlo method, because missing values were less than 0.5% in all variables except for C-reactive protein (295 of 6264; 4.71%). Categorical variables are reported as frequencies (percentages), and continuous variables are reported as medians with interquartile ranges. Differences between groups were evaluated using the chi-square test or Fisher’s exact test for categorical variables; and the independent t-test, one-way analysis of variance, Mann-Whitney rank-sum test, or Kruskal-Wallis test were used for continuous variables, as appropriate.

Unadjusted relationships between potential predictors and primary outcome were examined by univariate Cox proportional hazard models, and Kaplan-Meier curves were used for graphical presentation. For the continuous analysis, restricted cubic spline testing indicated there were significant non-linear relationships between serum aminotransferase levels and the primary outcome. Thus, the restricted cubic spline variables were also included in the final model. In the multivariable analyses, adjustments were made for potential confounders from the European System for Cardiac Operative Risk Evaluation (EuroSCORE) or the Society of Thoracic Surgeons risk models and from background knowledge such as sex, age, body mass index, estimated glomerular filtration rate, type of surgery, urgent surgery, ejection fraction, pulmonary hypertension, acute coronary syndrome, liver disease, statin, alcohol consumption, albumin, and bilirubin. Multicollinearity was assessed with the variance inflation factor using a reference value of four, and Schoenfeld residuals were used to check the proportionality assumption. For secondary outcomes, multivariable logistic regression models were used, and odds ratios were adjusted by the same possible confounders used in primary outcome analysis. We performed additional interaction analyses to determine possible effect modification by the prespecified subgroups (age ≤60 or >60 years and patients with or without liver disease). To verify the usefulness of serum aminotransferase as a new prognostic marker, incremental values incorporating serum aminotransferase levels into the main multivariable model were assessed. Net reclassification improvement and integrated discrimination improvement were used to quantify the incremental values [35].

Sensitivity analyses were performed to assess the selection biases from exposure definition. For this purpose, we categorized serum aminotransferase levels by two different methods (≤15th percentile, 15th–85th percentile, and >85th percentile; and K-Adaptive Partitioning for survival data [36]) and repeated the analysis. We performed another sensitivity analysis to verify the effects of other possible confounders. In these models, logistic EuroSCORE, MELD Xi (Model for End-stage Liver Disease eXcluding INR) [37] scores, and other variables found to be statistically significant (*P<*0.05) in the univariate analysis were included. The variables used for logistic EuroSCORE and MELD Xi calculations were excluded in this model.

All statistical analyses were performed with R version 3.5.1 (R Foundation for Statistical Computing, Vienna, Austria) and SAS version 9.4 (SAS Institute, Cary, NC). Two-sided *P* values <0.05 were considered statistically significant.

## Supplementary Material

Supplementary Table 1

Supplementary Table 2

## References

[1] Pratt DS, Kaplan MM. Evaluation of abnormal liver-enzyme results in asymptomatic patients. N Engl J Med. 2000; 342:1266–71. 10.1056/NEJM200004273421707 10781624

[2] Kunutsor SK, Apekey TA, Khan H. Liver enzymes and risk of cardiovascular disease in the general population: a meta-analysis of prospective cohort studies. Atherosclerosis. 2014; 236:7–17. 10.1016/j.atherosclerosis.2014.06.006 24998934

[3] Liu Z, Ning H, Que S, Wang L, Qin X, Peng T. Complex association between alanine aminotransferase activity and mortality in general population: a systematic review and meta-analysis of prospective studies. PLoS One. 2014; 9:e91410. 10.1371/journal.pone.0091410 24633141 PMC3954728

[4] Choi KM, Han K, Park S, Chung HS, Kim NH, Yoo HJ, Seo JA, Kim SG, Kim NH, Baik SH, Park YG, Kim SM. Implication of liver enzymes on incident cardiovascular diseases and mortality: A nationwide population-based cohort study. Sci Rep. 2018; 8:3764. 10.1038/s41598-018-19700-8 29491346 PMC5830612

[5] Rahimzadeh P, Safari S, Faiz SH, Alavian SM. Anesthesia for patients with liver disease. Hepat Mon. 2014; 14:e19881–19881. 10.5812/hepatmon.19881 25031586 PMC4080095

[6] Bishop MJ, Souders JE, Peterson CM, Henderson WG, Domino KB. Factors associated with unanticipated day of surgery deaths in Department of Veterans Affairs hospitals. Anesth Analg. 2008; 107:1924–35. 10.1213/ane.0b013e31818af8f3 19020140

[7] Møller S, Bernardi M. Interactions of the heart and the liver. Eur Heart J. 2013; 34:2804–11. 10.1093/eurheartj/eht246 23853073

[8] Nikolaou M, Parissis J, Yilmaz MB, Seronde MF, Kivikko M, Laribi S, Paugam-Burtz C, Cai D, Pohjanjousi P, Laterre PF, Deye N, Poder P, Cohen-Solal A, Mebazaa A. Liver function abnormalities, clinical profile, and outcome in acute decompensated heart failure. Eur Heart J. 2013; 34:742–49. 10.1093/eurheartj/ehs332 23091203

[9] Schmucker DL. Age-related changes in liver structure and function: implications for disease? Exp Gerontol. 2005; 40:650–59. 10.1016/j.exger.2005.06.009 16102930

[10] Elinav E, Ackerman Z, Maaravi Y, Ben-Dov IZ, Ein-Mor E, Stessman J. Low alanine aminotransferase activity in older people is associated with greater long-term mortality. J Am Geriatr Soc. 2006; 54:1719–24. 10.1111/j.1532-5415.2006.00921.x 17087699

[11] Le Couteur DG, Blyth FM, Creasey HM, Handelsman DJ, Naganathan V, Sambrook PN, Seibel MJ, Waite LM, Cumming RG. The association of alanine transaminase with aging, frailty, and mortality. J Gerontol A Biol Sci Med Sci. 2010; 65:712–7. 10.1093/gerona/glq082 20498223 PMC4085878

[12] Vespasiani-Gentilucci U, De Vincentis A, Ferrucci L, Bandinelli S, Antonelli Incalzi R, Picardi A. Low Alanine Aminotransferase Levels in the Elderly Population: Frailty, Disability, Sarcopenia, and Reduced Survival. J Gerontol A Biol Sci Med Sci. 2018; 73:925–30. 10.1093/gerona/glx126 28633440 PMC6001897

[13] Lee H, Lee SE, Byun SS, Kim HH, Kwak C, Hong SK. De Ritis ratio (aspartate transaminase/alanine transaminase ratio) as a significant prognostic factor after surgical treatment in patients with clear-cell localized renal cell carcinoma: a propensity score-matched study. BJU Int. 2017; 119:261–67. 10.1111/bju.13545 27226065

[14] Bezan A, Mrsic E, Krieger D, Stojakovic T, Pummer K, Zigeuner R, Hutterer GC, Pichler M. The Preoperative AST/ALT (De Ritis) Ratio Represents a Poor Prognostic Factor in a Cohort of Patients with Nonmetastatic Renal Cell Carcinoma. J Urol. 2015; 194:30–35. 10.1016/j.juro.2015.01.083 25623738

[15] Winter JM, Cameron JL, Yeo CJ, Alao B, Lillemoe KD, Campbell KA, Schulick RD. Biochemical markers predict morbidity and mortality after pancreaticoduodenectomy. J Am Coll Surg. 2007; 204:1029–36. 10.1016/j.jamcollsurg.2007.01.026 17481534

[16] Hughes C, Hurtuk MG, Rychlik K, Shoup M, Aranha GV. Preoperative liver function tests and hemoglobin will predict complications following pancreaticoduodenectomy. J Gastrointest Surg. 2008; 12:1822–27. 10.1007/s11605-008-0680-y 18787909

[17] Ramaty E, Maor E, Peltz-Sinvani N, Brom A, Grinfeld A, Kivity S, Segev S, Sidi Y, Kessler T, Sela BA, Segal G. Low ALT blood levels predict long-term all-cause mortality among adults. A historical prospective cohort study. Eur J Intern Med. 2014; 25:919–21. 10.1016/j.ejim.2014.10.019 25468741

[18] Koehler EM, Sanna D, Hansen BE, van Rooij FJ, Heeringa J, Hofman A, Tiemeier H, Stricker BH, Schouten JN, Janssen HL. Serum liver enzymes are associated with all-cause mortality in an elderly population. Liver Int. 2014; 34:296–304. 10.1111/liv.12311 24219360

[19] Oh CM, Won YJ, Cho H, Lee JK, Park BY, Jun JK, Koh DH, Ki M, Jung KW, Oh IH. Alanine aminotransferase and gamma-glutamyl transferase have different dose-response relationships with risk of mortality by age. Liver Int. 2016; 36:126–35. 10.1111/liv.12879 26036985

[20] Ambrosy AP, Dunn TP, Heidenreich PA. Effect of minor liver function test abnormalities and values within the normal range on survival in heart failure. Am J Cardiol. 2015; 115:938–41. 10.1016/j.amjcard.2015.01.023 25708860

[21] Williams KH, Sullivan DR, Nicholson GC, George J, Jenkins AJ, Januszewski AS, Gebski VJ, Manning P, Tan YM, Donoghoe MW, Ehnholm C, Young S, O’Brien R, et al. Opposite associations between alanine aminotransferase and γ-glutamyl transferase levels and all-cause mortality in type 2 diabetes: Analysis of the Fenofibrate Intervention and Event Lowering in Diabetes (FIELD) study. Metabolism. 2016; 65:783–93. 10.1016/j.metabol.2015.12.008 27085785

[22] Kim IH, Kisseleva T, Brenner DA. Aging and liver disease. Curr Opin Gastroenterol. 2015; 31:184–91. 10.1097/MOG.0000000000000176 25850346 PMC4736713

[23] Irina G, Refaela C, Adi B, Avia D, Liron H, Chen A, Gad S. Low Blood ALT Activity and High FRAIL Questionnaire Scores Correlate with Increased Mortality and with Each Other. A Prospective Study in the Internal Medicine Department. J Clin Med. 2018; 7:386. 10.3390/jcm7110386 30366377 PMC6262457

[24] Pincus MR, Tierno PM, Gleeson E, Bowne WB, Bluth MH (McPherson RA, Pincus MR, editors). Evaluation of Liver Function, Henry’s clinical diagnosis and management by laboratory methods. 23rd ed. St. Louis (Missouri): Elsevier; 2017 pp. 289–305.

[25] Mythili S, Malathi N. Diagnostic markers of acute myocardial infarction. Biomed Rep. 2015; 3:743–48. 10.3892/br.2015.500 26623010 PMC4660641

[26] Koh LY, Hwang NC. Frailty in Cardiac Surgery. J Cardiothorac Vasc Anesth. 2019; 33:521–31. 10.1053/j.jvca.2018.02.032 29580797

[27] Gilbert T, Neuburger J, Kraindler J, Keeble E, Smith P, Ariti C, Arora S, Street A, Parker S, Roberts HC, Bardsley M, Conroy S. Development and validation of a Hospital Frailty Risk Score focusing on older people in acute care settings using electronic hospital records: an observational study. Lancet. 2018; 391:1775–82. 10.1016/S0140-6736(18)30668-8 29706364 PMC5946808

[28] Rothenberg DM, O’Connor CJ, Tuman KJ. Anesthesia and the hepatobiliary system, Miller’s Anesthesia, eighth edition. Edited by Miller RoD. Philadelphia, PA, Saunders, an imprint of Elsevier Inc, 2015; pp 2244–61.

[29] Dhillon A, Steadman RH. Liver disease, Anesthesia and Uncommon Diseases, sixth edition. Edited by Fleisher LA. Philadelphia, PA, Saunders, an imprint of Elsevier Inc, 2012; pp 162–214. 10.1016/B978-1-4377-2787-6.00005-X

[30] von Elm E, Altman DG, Egger M, Pocock SJ, Gøtzsche PC, Vandenbroucke JP, and STROBE Initiative. The Strengthening the Reporting of Observational Studies in Epidemiology (STROBE) Statement: guidelines for reporting observational studies. Int J Surg. 2014; 12:1495–99. 10.1016/j.ijsu.2014.07.013 25046131

[31] Joung KW, Jo JY, Kim WJ, Choi DK, Chin JH, Lee EH, Choi IC. Association of preoperative uric acid and acute kidney injury following cardiovascular surgery. J Cardiothorac Vasc Anesth. 2014; 28:1440–47. 10.1053/j.jvca.2014.04.020 25245579

[32] Jammer I, Wickboldt N, Sander M, Smith A, Schultz MJ, Pelosi P, Leva B, Rhodes A, Hoeft A, Walder B, Chew MS, Pearse RM, and European Society of Anaesthesiology (ESA), and the European Society of Intensive Care Medicine (ESICM), and European Society of Anaesthesiology, and European Society of Intensive Care Medicine. Standards for definitions and use of outcome measures for clinical effectiveness research in perioperative medicine: European Perioperative Clinical Outcome (EPCO) definitions: a statement from the ESA-ESICM joint taskforce on perioperative outcome measures. Eur J Anaesthesiol. 2015; 32:88–105. 10.1097/EJA.0000000000000118 25058504

[33] Lee EH, Yun SC, Chin JH, Choi DK, Son HJ, Kim WC, Choi SS, Song JG, Hahm KD, Sim JY, Choi IC. Prognostic implications of preoperative E/e′ ratio in patients with off-pump coronary artery surgery. Anesthesiology. 2012; 116:362–71. 10.1097/ALN.0b013e3182426ed6 22222471

[34] Shahian DM, Jacobs JP, Badhwar V, Kurlansky PA, Furnary AP, Cleveland JC Jr, Lobdell KW, Vassileva C, Wyler von Ballmoos MC, Thourani VH, Rankin JS, Edgerton JR, D’Agostino RS, et al. The Society of Thoracic Surgeons 2018 Adult Cardiac Surgery Risk Models: Part 1-Background, Design Considerations, and Model Development. Ann Thorac Surg. 2018; 105:1411–18. 10.1016/j.athoracsur.2018.03.002 29577925

[35] Uno H, Tian L, Cai T, Kohane IS, Wei LJ. A unified inference procedure for a class of measures to assess improvement in risk prediction systems with survival data. Stat Med. 2013; 32:2430–42. 10.1002/sim.5647 23037800 PMC3734387

[36] Eo SH, Kang HJ, Hong SM, Cho H. K-Adaptive Partitioning for Survival Data, with an Application to Cancer Staging. arXiv e-prints. 2013.

[37] Heuman DM, Mihas AA, Habib A, Gilles HS, Stravitz RT, Sanyal AJ, Fisher RA. MELD-XI: a rational approach to “sickest first” liver transplantation in cirrhotic patients requiring anticoagulant therapy. Liver Transpl. 2007; 13:30–37. 10.1002/lt.20906 17154400

