# Supplementary material for: Age-dependent relationship between preoperative serum aminotransferase and mortality after cardiovascular surgery
Source: Aging (Albany NY). 2019 Oct 18;11(20):9060–74. doi: 10.18632/aging.102374 (PMC6834416; doi:10.18632/aging.102374)
Supplement: Supplementary Table 1 [file aging-11-102374-s001.docx]

**Supplementary Table 1. Patient characteristics according to aminotransferase groups**

|  |  | **ALT** |  |  | **AST** |  |  | **De Ritis ratio** |  |
| --- | --- | --- | --- | --- | --- | --- | --- | --- | --- |
|  | Low  (*n =* 1528) | Middle  (*n =* 3512) | High  (*n =* 1224) | Low  (*n =* 1384) | Middle  (*n =* 3687) | High  (*n =* 1193) | Low  (*n =* 1274) | Middle  (*n =* 3745) | High  (*n =* 1245) |
| 90-day mortality | 77 (5.0) | 80 (2.3) | 26 (2.1) | 46 (3.3) | 85 (2.3) | 52 (4.4) | 17 (1.3) | 87 (2.3) | 79 (6.3) |
| ALT (IU/L) | 11.0  [9.0–12.0] | 19.0  [16.0–23.0] | 41.0  [34.0–54.0] | 12.0  [10.0–16.0] | 19.0  [15.0–25.0] | 36.0  [24.0–51.0] | 34.0  [26.0–49.0] | 18.0  [15.0–24.0] | 12.0  [9.0–15.0] |
| AST (IU/L) | 17.0  [15.0–20.0] | 22.0  [19.0–27.0] | 33.0  [27.0–43.0] | 15.0  [14.0–16.0] | 22.0  [20.0–25.0] | 38.0  [33.0–48.0] | 23.0  [19.0–31.0] | 22.0  [18.0–27.0] | 23.0  [18.0–30.0] |
| De Ritis ratio | 1.7  [1.4– 2.0] | 1.1  [1.0– 1.4] | 0.8  [0.6– 0.9] | 1.2  [0.9– 1.5] | 1.2  [0.9– 1.5] | 1.1  [0.8– 1.7] | 0.7  [0.6– 0.8] | 1.2  [1.0– 1.4] | 1.9  [1.8– 2.2] |
| Age (years) | 64.0  [53.0–72.0] | 62.0  [53.0–70.0] | 58.0  [50.0–67.0] | 61.0  [49.0–70.0] | 62.0  [53.0–70.0] | 62.0  [53.0–70.0] | 57.0  [48.0–65.0] | 62.0  [53.0–70.0] | 65.0  [55.0–72.0] |
| Female sex | 845 (55.3) | 1370 (39.0) | 315 (25.7) | 582 (42.1) | 1509 (40.9) | 439 (36.8) | 247 (19.4) | 1575 (42.1) | 708 (56.9) |
| BMI (kg/m^2^) | 22.9  [20.8–25.0] | 24.0  [21.9–26.1] | 24.8  [22.5–27.0] | 23.6  [21.4–25.8] | 24.0  [21.9–26.1] | 23.8  [21.6–26.2] | 25.1  [23.1–27.2] | 23.9  [21.9–26.0] | 22.5  [20.4–24.6] |
| Alcohol | 41 (2.7) | 158 (4.5) | 65 (5.3) | 62 (4.5) | 136 (3.7) | 66 (5.5) | 67 (5.3) | 153 (4.1) | 44 (3.5) |
| Type of surgery |  |  |  |  |  |  |  |  |  |
| CABG | 288 (18.8) | 982 (28.0) | 461 (37.7) | 362 (26.2) | 999 (27.1) | 370 (31.0) | 506 (39.7) | 1000 (26.7) | 225 (18.1) |
| Valve | 672 (44.0) | 1617 (46.0) | 494 (40.4) | 517 (37.4) | 1700 (46.1) | 566 (47.4) | 470 (36.9) | 1693 (45.2) | 620 (49.8) |
| Aorta | 157 (10.3) | 195 (5.6) | 47 (3.8) | 154 (11.1) | 199 (5.4) | 46 (3.9) | 74 (5.8) | 220 (5.9) | 105 (8.4) |
| Combined | 323 (21.1) | 580 (16.5) | 177 (14.5) | 266 (19.2) | 636 (17.2) | 178 (14.9) | 167 (13.1) | 666 (17.8) | 247 (19.8) |
| Other | 88 (5.8) | 138 (3.9) | 45 (3.7) | 85 (6.1) | 153 (4.1) | 33 (2.8) | 57 (4.5) | 166 (4.4) | 48 (3.9) |
| Urgent surgery | 46 (3.0) | 87 (2.5) | 59 (4.8) | 50 (3.6) | 76 (2.1) | 66 (5.5) | 45 (3.5) | 101 (2.7) | 46 (3.7) |
| ACS | 122 (8.0) | 411 (11.7) | 220 (18.0) | 130 (9.4) | 406 (11.0) | 217 (18.2) | 198 (15.5) | 423 (11.3) | 132 (10.6) |
| Inotrope/vasopressor | 61 (4.0) | 111 (3.2) | 41 (3.3) | 43 (3.1) | 108 (2.9) | 62 (5.2) | 35 (2.7) | 112 (3.0) | 66 (5.3) |
| Atrial fibrillation | 290 (19.0) | 824 (23.5) | 246 (20.1) | 135 (9.8) | 873 (23.7) | 352 (29.5) | 177 (13.9) | 806 (21.5) | 377 (30.3) |
| Ejection fraction (%) | 61.0  [55.0–65.0] | 60.0  [55.0–65.0] | 60.0  [51.0–64.0] | 61.0  [56.0–65.0] | 61.0  [55.0–65.0] | 59.0  [50.0–64.0] | 60.0  [54.0–65.0] | 61.0  [55.0–65.0] | 60.0  [53.5–65.0] |
| Pulmonary hypertension | 504 (33.1) | 1008 (28.8) | 348 (28.5) | 319 (23.2) | 1084 (29.6) | 457 (38.3) | 273 (21.6) | 1056 (28.3) | 531 (42.9) |
| Diabetes mellitus | 352 (23.0) | 860 (24.5) | 321 (26.2) | 398 (28.8) | 841 (22.8) | 294 (24.6) | 373 (29.3) | 896 (23.9) | 264 (21.2) |
| Hypertension | 748 (49.0) | 1724 (49.1) | 592 (48.4) | 740 (53.5) | 1760 (47.7) | 564 (47.3) | 652 (51.2) | 1832 (48.9) | 580 (46.6) |
| Dyslipidemia | 1056 (69.1) | 2637 (75.1) | 931 (76.1) | 1025 (74.1) | 2743 (74.4) | 856 (71.8) | 993 (77.9) | 2805 (74.9) | 826 (66.3) |
| Heart failure | 128 (8.4) | 230 (6.5) | 101 (8.3) | 74 (5.3) | 253 (6.9) | 132 (11.1) | 87 (6.8) | 230 (6.1) | 142 (11.4) |
| Coronary revascularization | 111 (7.3) | 360 (10.3) | 102 (8.3) | 128 (9.2) | 334 (9.1) | 111 (9.3) | 122 (9.6) | 343 (9.2) | 108 (8.7) |
| Hepatic disease | 74 (4.8) | 180 (5.1) | 90 (7.4) | 40 (2.9) | 178 (4.8) | 126 (10.6) | 68 (5.3) | 166 (4.4) | 110 (8.8) |
| Albumin (g/L) | 37  [34– 40] | 38  [35– 41] | 38  [34– 41] | 37  [34– 40] | 38  [35– 41] | 37  [33– 40] | 38  [35– 41] | 38  [35– 41] | 37  [33– 39] |
| Bilirubin (µmol/L) | 10.3  [6.8– 13.7] | 10.3  [6.8– 15.4] | 10.3  [8.6– 15.4] | 8.6  [6.8– 12.0] | 10.3  [6.8– 15.4] | 12.0  [8.6– 18.8] | 10.3  [6.8– 13.7] | 10.3  [6.8– 15.4] | 12.0  [8.6– 17.1] |
| Hematocrit (%) | 36.4  [32.6–39.6] | 39.2  [35.9–42.2] | 40.1  [36.0–43.4] | 37.8  [33.8–40.7] | 39.1  [35.7–42.2] | 38.7  [34.1–42.3] | 40.7  [37.0–43.6] | 38.9  [35.7–41.9] | 35.7  [31.8–39.5] |
| eGFR (mL/min/1.73m^2^) | 84.1  [62.0–96.8] | 87.3  [71.6–97.7] | 88.3  [72.9–99.4] | 87.4  [67.3–99.7] | 86.9  [71.4–97.6] | 85.6  [68.2–97.1] | 89.9  [75.7–100.8] | 86.8  [70.9–97.7] | 82.4  [61.6–94.3] |
| Sodium (mmol/L) | 140  [138–142] | 140  [138–142] | 140  [138–141] | 140  [139–142] | 140  [138–142] | 139  [137–141] | 140  [138–142] | 140  [138–142] | 139  [137–141] |
| Uric acid (µmol/L) | 315  [250– 393] | 327  [268– 393] | 339  [274– 405] | 315  [250– 387] | 327  [268– 393] | 339  [274– 416] | 339  [274– 405] | 321  [262– 393] | 321  [256– 410] |
| CRP (nmol/L) | 0.95  [0.95– 5.7] | 0.95  [0.95– 2.9] | 1.9  [0.95– 5.7] | 0.95  [0.95– 4.8] | 0.95  [0.95– 2.9] | 1.9  [0.95– 6.7] | 0.95  [0.95– 3.8] | 0.95  [0.95– 2.9] | 1.9  [0.95– 6.7] |
| Statin | 602 (39.4) | 1731 (49.3) | 648 (52.9) | 646 (46.7) | 1763 (47.8) | 572 (47.9) | 711 (55.8) | 1803 (48.1) | 467 (37.5) |
| Diuretics | 685 (44.8) | 1547 (44.0) | 501 (40.9) | 495 (35.8) | 1615 (43.8) | 623 (52.2) | 456 (35.8) | 1582 (42.2) | 695 (55.8) |
| EuroSCORE (logistic) | 4.8  [2.5– 9.7] | 3.3  [1.9– 6.5] | 2.9  [1.5– 5.8] | 4.0  [2.1– 7.2] | 3.4  [1.9– 6.6] | 4.0  [2.1– 8.6] | 2.4  [1.5– 4.6] | 3.5  [2.1– 6.6] | 5.5  [3.1–11.1] |
| MELD Xi | 9.0  [9.0–11.0] | 9.0  [9.0–11.0] | 9.0  [9.0–11.0] | 9.0  [9.0–11.0] | 9.0  [9.0–11.0] | 9.0  [9.0–12.0] | 9.0  [9.0–10.0] | 9.0  [9.0–11.0] | 9.0  [9.0–12.0] |

Data are expressed as the number of patients (%) and mean (±standard deviation) or median [interquartile range].

SI conversion factors: To convert ALT and AST to µkat/L, multiply values by 0.0167.

ALT = alanine aminotransferase; AST = aspartate aminotransferase; BMI = body mass index; CABG = coronary artery bypass graft; ACS = acute coronary syndrome; eGFR = estimated glomerular filtration rate; CRP = C-reactive protein; EuroSCORE = The European System for Cardiac Operative Risk Evaluation; MELD Xi = Model for End-stage Liver Disease.
