# Supplementary material for: Age-dependent relationship between preoperative serum aminotransferase and mortality after cardiovascular surgery
Source: Aging (Albany NY). 2019 Oct 18;11(20):9060–74. doi: 10.18632/aging.102374 (PMC6834416; doi:10.18632/aging.102374)
Supplement: Supplementary Table 2 [file aging-11-102374-s002.docx]

**Supplementary Table 2. Definitions of outcomes and variables.**

|  | **Definitions** |
| --- | --- |
| **Secondary outcomes** |  |
| In-hospital death | All-cause death during the hospital stay or within postoperative day 30 [1] |
| Prolonged hospital length of stay | Length of hospital stay >14 days [1] |
| Major adverse cardiovascular events | Cardiac arrest, myocardial infarction, low cardiac output syndrome, use of mechanical assist devices, readmission due to heart failure, ventricular tachycardia, or ventricular fibrillation within postoperative day 30 |
| Pulmonary complications | Mechanical ventilation ≥48 hours, unplanned re-intubation, pneumonia, or acute lung injury within postoperative day 30 |
| Acute kidney injury | Acute kidney injury according to KDIGO (Kidney Disease Improving Global Outcomes) criteria stage ≥2 within postoperative day 30 [2] |
| Stroke | Ischemic stroke as defined by the Neurologic Academic Research Consortium (NeuroARC) class Ia within postoperative day 30 [3] |
| Gastrointestinal complications | Abdominal organ ischemia, acute hepatitis/pancreatitis/cholecystitis, intestinal perforation/bleeding, and bowel obstruction within postoperative day 30 |
| **Variables** |  |
| Age (years) | Age of patients at the time of surgery |
| Female sex | Number of female sex patients |
| Body mass index (kg/m^2^) | Body mass divided by the square of the body height |
| Diabetes mellitus | Diabetes mellitus requiring oral medication or insulin |
| Hypertension | Hypertension requiring anti-hypertensive medication |
| Dyslipidemia | Dyslipidemia requiring lipid-lowering medication or preoperative low-density lipoprotein 100 mg/dL or higher |
| Congestive heart failure | Symptomatic congestive heart failure |
| Coronary revascularization | History of coronary artery bypass or percutaneous coronary intervention |
| Liver disease | Liver cirrhosis of any cause, chronic viral hepatitis, toxic hepatitis, non-alcoholic fatty liver disease |
| Alcohol | Alcohol >2 drinks/day in 2 weeks before admission (according to American College of Surgeons National Surgical Quality Improvement Program) [4] |
| Acute coronary syndrome | Acute myocardial infarction within two weeks before surgery or resting chest pain at the time of surgery |
| Atrial fibrillation | Persistent or paroxysmal atrial fibrillation/flutter |
| EuroSCORE (logistic) | Predicted mortality according to the logistic regression model of EuroSCORE [5] |
| MELD Xi | Model for End-stage Liver Disease eXcluding INR score [6] |
| Ejection fraction (%) | Ejection fraction on preoperative echocardiography |
| Pulmonary hypertension | Mean pulmonary artery pressure ≥25 mmHg assessed by right heart catheterization, peak tricuspid regurgitation velocity ≥2.9 m/s or early diastolic pulmonary regurgitation velocity >2.2 m/s on preoperative echocardiography [7] |
| Hematocrit (%) | Preoperative hematocrit |
| Creatinine (mg/dL) | Preoperative serum creatinine |
| eGFR (mL/min/1.73 m^2^) | Estimated glomerular filtration rate according to Chronic Kidney Disease Epidemiology Collaboration (CKD-EPI) Equation [8] |
| Albumin (g/dL) | Preoperative serum albumin |
| Bilirubin (mg/dL) | Preoperative serum total bilirubin |
| Sodium (mmol/L) | Preoperative serum sodium |
| Uric acid (mg/dL) | Preoperative serum uric acid |
| C-reactive protein (mg/dL) | Preoperative serum C-reactive protein |
| ACEI or ARB | Patients with ACEI or ARB medications |
| Beta blocker | Patients with beta blocker medications |
| Calcium channel blocker | Patients with calcium channel blocker medications |
| Diuretics | Patients with diuretics medications |
| Statin | Patients with statin medications |
| Insulin | Patients with insulin medications |
| Inotropes/vasopressors | Preoperative inotropes or vasopressors use |
| Type of surgery | Types of cardiovascular surgery; coronary artery bypass graft, isolated cardiac valve surgery, other intracardiac surgery, thoracic aortic surgery, or combined procedure of the above operations |
| Urgent surgery | Patients not electively admitted for operation but who require intervention or surgery on the current admission for medical reasons [9] |
| Off-pump surgery | Surgery without cardiopulmonary bypass |
| Operation time (min) | Duration of operation |
| CPB time (min) | Duration of cardiopulmonary bypass |

EuroSCORE = The European System for Cardiac Operative Risk Evaluation; MELD Xi = Model for End-stage Liver Disease eXcluding INR; eGFR = estimated glomerular filtration rate; ACEI = angiotensin-converting enzyme inhibitor; ARB = angiotensin receptor blocker; CPB = Cardiopulmonary bypass.

1. Shahian DM, Jacobs JP, Badhwar V, Kurlansky PA, Furnary AP, Cleveland JC Jr, Lobdell KW, Vassileva C, Wyler von Ballmoos MC, Thourani VH, Rankin JS, Edgerton JR, D'Agostino RS, et al. The Society of Thoracic Surgeons 2018 Adult Cardiac Surgery Risk Models: Part 1-Background, Design Considerations, and Model Development. The Annals of Thoracic Surgery. 2018; 105:1411–1418.

<https://doi.org/10.1016/j.athoracsur.2018.03.002> [PMID:29577925](https://www.ncbi.nlm.nih.gov/pubmed/29577925)

1. Khwaja A. KDIGO Clinical Practice Guidelines for Acute Kidney Injury. Nephron Clinical Practice. 2012; 120:c179–c184.

<https://doi.org/10.1159/000339789> [PMID:22890468](https://www.ncbi.nlm.nih.gov/pubmed/22890468)

1. Lansky AJ, Messe SR, Brickman AM, Dwyer M, van der Worp HB, Lazar RM, Pietras CG, Abrams KJ, McFadden E, Petersen NH, Browndyke J, Prendergast B, Ng VG, et al. Proposed Standardized Neurological Endpoints for Cardiovascular Clinical Trials: An Academic Research Consortium Initiative. Journal of the American College of Cardiology. 2017; 69:679–691.

<https://doi.org/10.1016/j.jacc.2016.11.045> [PMID:28183511](https://www.ncbi.nlm.nih.gov/pubmed/28183511)

1. Temraz S, Tamim H, Mailhac A, and Taher A. Could sodium imbalances predispose to postoperative venous thromboembolism? An analysis of the NSQIP database. Thrombosis Journal. 2018; 16:11. <https://doi.org/10.1186/s12959-018-0165-5> [PMID:29988709](https://www.ncbi.nlm.nih.gov/pubmed/29988709)
2. Roques F, Michel P, Goldstone AR, and Nashef SAM. The logistic EuroSCORE. European Heart Journal. 2003; 24:882–883.

<https://doi.org/10.1016/S0195-668X(02)00799-6>

1. Heuman DM, Mihas AA, Habib A, Gilles HS, Stravitz RT, Sanyal AJ, and Fisher RA. MELD-XI: a rational approach to "sickest first" liver transplantation in cirrhotic patients requiring anticoagulant therapy. Liver Transplantation. 2007; 13:30–37.

<https://doi.org/10.1002/lt.20906> [PMID:17154400](https://www.ncbi.nlm.nih.gov/pubmed/17154400)

1. Torbicki A, Peacock A, Vonk Noordegraaf A, Ghofrani A, Hansmann G, Simonneau G, Lang I, Vachiery J-L, Pierard LA, Matucci M, Hoeper M, Beghetti M, Zompatori M, et al. 2015 ESC/ERS Guidelines for the diagnosis and treatment of pulmonary hypertension: The Joint Task Force for the Diagnosis and Treatment of Pulmonary Hypertension of the European Society of Cardiology (ESC) and the European Respiratory Society (ERS): Endorsed by: Association for European Paediatric and Congenital Cardiology (AEPC), International Society for Heart and Lung Transplantation (ISHLT). European Heart Journal. 2015; 37:67–119. <https://doi.org/10.1093/eurheartj/ehv317> [PMID:26320113](https://www.ncbi.nlm.nih.gov/pubmed/26320113)
2. Levey AS, Stevens LA, Schmid CH, Zhang YL, Castro AF, 3rd, Feldman HI, Kusek JW, Eggers P, Van Lente F, Greene T, and Coresh J. A New Equation to Estimate Glomerular Filtration Rate. Annals of Internal Medicine. 2009; 150:604–612.

<https://doi.org/10.7326/0003-4819-150-9-200905050-00006> [PMID:19414839](https://www.ncbi.nlm.nih.gov/pubmed/19414839)

1. Smith C, Nashef SAM, Roques F, Sharples LD, Nilsson J, Goldstone AR, and Lockowandt U. EuroSCORE II. European Journal of Cardio-Thoracic Surgery. 2012; 41:734–745.

<https://doi.org/10.1093/ejcts/ezs043> [PMID:22378855](https://www.ncbi.nlm.nih.gov/pubmed/22378855)
